# A novel homozygous intronic variant in *CDT1* that alters splicing causes Meier–Gorlin syndrome, and a review of published mutations and growth hormone treatments

**DOI:** 10.1186/s13023-024-03430-4

**Published:** 2024-12-18

**Authors:** Qing Li, Yichi Wu, Fucheng Meng, Zhuxi Li, Di Zhan, Xiaoping Luo

**Affiliations:** 1 Department of Pediatrics, Tongji Hospital, Tongji Medical College, Huazhong University of Science and Technology, Wuhan, China; 2 Hubei Key Laboratory of Pediatric Genetic Metabolic and Endocrine Rare Diseases, Wuhan, China

**Keywords:** Meier–Gorlin syndrome, *CDT1*, Intronic mutation, Short stature, Growth hormone treatment

## Abstract

**Background:**

Meier–Gorlin syndrome (MGORS) is a rare autosomal inherited form of primordial dwarfism. Pathogenic variants in 13 genes involved in DNA replication initiation have been identified in this disease, but homozygous intronic variants have never been reported. Additionally, whether growth hormone (GH) treatment can increase the height of children with MGORS is unclear.

**Methods:**

The medical history data of a young girl were collected and reviewed. Whole-exome sequencing (WES) and bioinformatic analysis were performed to identify any variants and predict their pathogenicity. Minigene constructs were generated and transfected into HEK-293T cells for in vitro splicing assays. The literature was reviewed to explore the mutational spectrum and efficacy of GH treatment for this disease.

**Results:**

A girl with microtia, hypoplastic patellae, and severe growth retardation carried a novel homozygous intronic variant (NM_030928.4: exon 3: c.352–30 A > C) in *CDT1*. The variant was predicted to break a branch point and alter splicing, and the minigene assay confirmed abnormal splicing with exon 3 skipping. The patient was treated with GH for 5 years, with an increase in growth velocity from 4.0 cm/year to an average of 6.2 cm/year. A literature review revealed that the most common variant type and inheritance state were missense and compound heterozygous, respectively. Additionally, the vast majority of children with MGORS treated with GH had normal insulin-like growth factor 1 (IGF-1) levels, and half of them responded positively to GH therapy.

**Conclusions:**

We reported a novel pathogenic homozygous intronic variant (c.352–30 A > C) of *CDT1* in a girl with MGORS, and this mutation extended the genetic spectrum of the disease. GH therapy may be beneficial for height outcomes in children with MGORS with normal IGF-1 levels.

**Supplementary Information:**

The online version contains supplementary material available at 10.1186/s13023-024-03430-4.

## Background

Meier–Gorlin syndrome (MGORS) is a rare autosomal inherited form of primordial dwarfism characterized by the triad of microtia, absent or hypoplastic patellae, and severe prenatal and postnatal growth retardation [1; 2]. The exact incidence of MGORS has not been determined, and fewer than 150 cases have been reported worldwide. The characteristic cephalic and facial deformities of MGORS include microtia, micrognathia, a high forehead, a narrow and convex nose, and a small mouth with thick lips. Other common features include feeding problems, microcephaly, mammary hypoplasia, external genital abnormalities, congenital pulmonary emphysema, and skeletal abnormalities [1]. Current treatments for the disease focus mainly on relieving related clinical symptoms. Only a few patients have been treated with GH to improve severe short stature, but its therapeutic efficacy is controversial [2–7]. Pathogenic variants in genes encoding highly conserved components involved in the initiation of DNA replication cause this disease. To date, 13 genes (*ORC1*, *ORC4*, *ORC6*, *CDT1*, *CDC6*, *CDC45*, *GMNN*, *MCM3*, *MCM5*, *MCM7*, *GINS2*, *GINS3*, and *DONSON*) have been reported to be responsible for the development of MGORS [8–11]. Most pathogenic mutations of MGORS are inherited in a compound heterozygous state [2; 10–12], and no homozygous intronic variant has been found yet.

Pre-messenger RNA (pre-mRNA) splicing, in which introns are removed and exons are strictly joined, is a primary step in protein expression. Mutations that interfere with normal mRNA splicing are critical contributors to rare genetic diseases [13; 14]. Canonical splice site (CSS) variants within 2 base pairs of the junctions of introns and exons are strong diagnostic candidates for genetic diseases, whereas the effects of noncanonical splicing variations on genetic disorders, including variants in branchpoints (BPs) and deep intronic regions (more than 100 bases far from the exon‒intron junction), are becoming increasingly recognized [15–17]. According to the estimate by Lord et al. [18], which is based on exomic sequencing data from approximately 8,000 patients, up to 27% of mutations are located at noncanonical positions. Furthermore, 48 genetic variants in the human genome have been reported to cause diseases by disrupting BPs to alter splicing [19].

MGORS is a congenital genetic disorder in which more than 80% of patients have a typical triad and need multidisciplinary management from birth to adulthood [1]. Microtia may lead to hearing loss, and ear–nose–throat specialist assistance is required. In very few severe cases, patellar hypoplasia may lead to mobility difficulties, and knee surgeries are needed. Only a few patients have been treated with GH to increase their height. GH treatments for MGORS patients with low levels of IGF-1 have been recommended by some researchers [1; 3]. However, the levels of IGF-1 are normal in more than 80% of patients with MGORS, and the therapeutic effect of GH in these patients remains unclear.

Here, we report the case of a girl with MGORS carrying a novel variant (c.352–30 A > C) in *CDT1*, making her the first known patient with MGORS caused by a homozygous intronic variant. We confirmed the pathogenicity of this variant via in vitro splicing assays. Five years of treatment with long-acting PEGylated recombinant human GH (PEG-rhGH) increased her height despite a consistently normal IGF-1 level. Moreover, the literature was reviewed to gain further insight into mutational features and whether GH treatment is helpful for height outcomes in children with MGORS.

## Methods

### Patient enrolment and growth data analysis

This study enrolled a 4-year-old Chinese girl with severe short stature from the Department of Paediatrics, Tongji Hospital. The clinical data and medical history of this patient were collected. Standard deviation (SDS) values for height, weight, and head circumference were calculated using Chinese 2009 growth data [20]. The mid-parental height (MPH) of the patient was calculated as (mother’s height + father’s height − 13 cm)/2 [21]. A combined insulin–clonidine stimulation experiment was carried out to evaluate the peak level of GH secretion, and a stimulated GH level ≥ 10 ng/mL was considered normal [22].

## Genetic testing and bioinformatic analysis

The peripheral blood samples of the child and her parents were collected in EDTA-containing tubes, and genomic DNA was extracted utilizing a QIAamp DNA Blood Mini Kit (Qiagen, Hilden, Germany) following the manufacturer’s recommendations. The genomic DNA was fragmented into 100–300 bp segments. xGen Exome Research Panel probes (IDT, USA) were used to capture the libraries, and the libraries were subsequently sequenced on the Illumina NovaSeq 6000 Sequencing platform. After quality control, the purified clean reads were mapped to the human reference genome (GRCh38/hg38). Variants were analysed by GATK-HaplotypeCaller and further annotated via public databases (the 1000 Genomes Project, gnomAD, ANNOVAR, and bSNP) [23]. The potentially pathogenic variants were confirmed by Sanger sequencing and evaluated according to the American College of Medical Genetics and Genomics (ACMG) guidelines [15]. Bioinformatic analysis tools, such as Human Splicing Finder (HSF), Splice AI, and dbscSNV, were used to assess the possible pathogenic effects of the splice site variants [24–26].

## Minigene plasmid construction

The fragment covering exons 2–4 of *CDT1* was amplified by nested primers to generate the wild-type and mutant fragments. The wild-type and mutated fragments were cloned and inserted into pECMV vectors to produce pECMV-CDT1-wild-type (wt) and pECMV-CDT1 mutant (mut) minigene constructs, respectively. Sanger sequencing was performed to confirm the sequence of the wt and mut fragments on the vectors. The primers used for the above operations are listed in Table S1. The pECMV vector was kindly donated by Cipher Gene.

## In vitro splicing assay

The minigene constructs were transferred into human embryonic kidney 293T (HEK-293T) cells by using Lipo3000 Reagent (Invitrogen, USA) according to the manufacturer’s recommendations. After 24 h, RNA from the wt and mut constructs was extracted from the transfected cells utilizing RNA-Easy Isolation Reagent (Vazyme, China), and cDNA was synthesized via reverse transcription with an equal amount of RNA. The primers at both ends of the minigene vector were used for PCR amplification. The amplified transcription bands were detected by agarose gel electrophoresis, and each band was recovered for Sanger sequencing.

## Literature review

The pathogenic variants of 13 MGORS-related genes were searched in the Human Gene Mutation Database (HGMD), and all the articles on MGORS carrying pathogenic variants or with GH treatment were searched in PubMed and a Chinese public database (China National Knowledge Infrastructure). The search terms for mutations were as follows: Meier–Gorlin syndrome, *ORC1*, *ORC4*, *ORC6*, *CDT1*, *CDC6*, *CDC45*, *GMNN*, *MCM3*, *MCM5*, *MCM7*, *GINS2*, *GINS3*, *DONSON*, primordial dwarfism, or ear-patella-short stature syndrome. Genetic information was collected, and patients without MGORS-associated clinical phenotypes were excluded. The search terms for GH treatment were as follows: Meier–Gorlin syndrome, growth hormone treatment, growth hormone therapy, recombinant growth hormone, or ear-patella-short stature syndrome. The clinical data of all patients receiving GH treatment were collected at the beginning of the treatment (including sex, mutated gene, height SDS, growth velocity, bone age, and IGF-1 levels) and at the last follow-up (including age, height SDS, and growth velocity).

## Results

### Clinical history

**Fig. 1:**
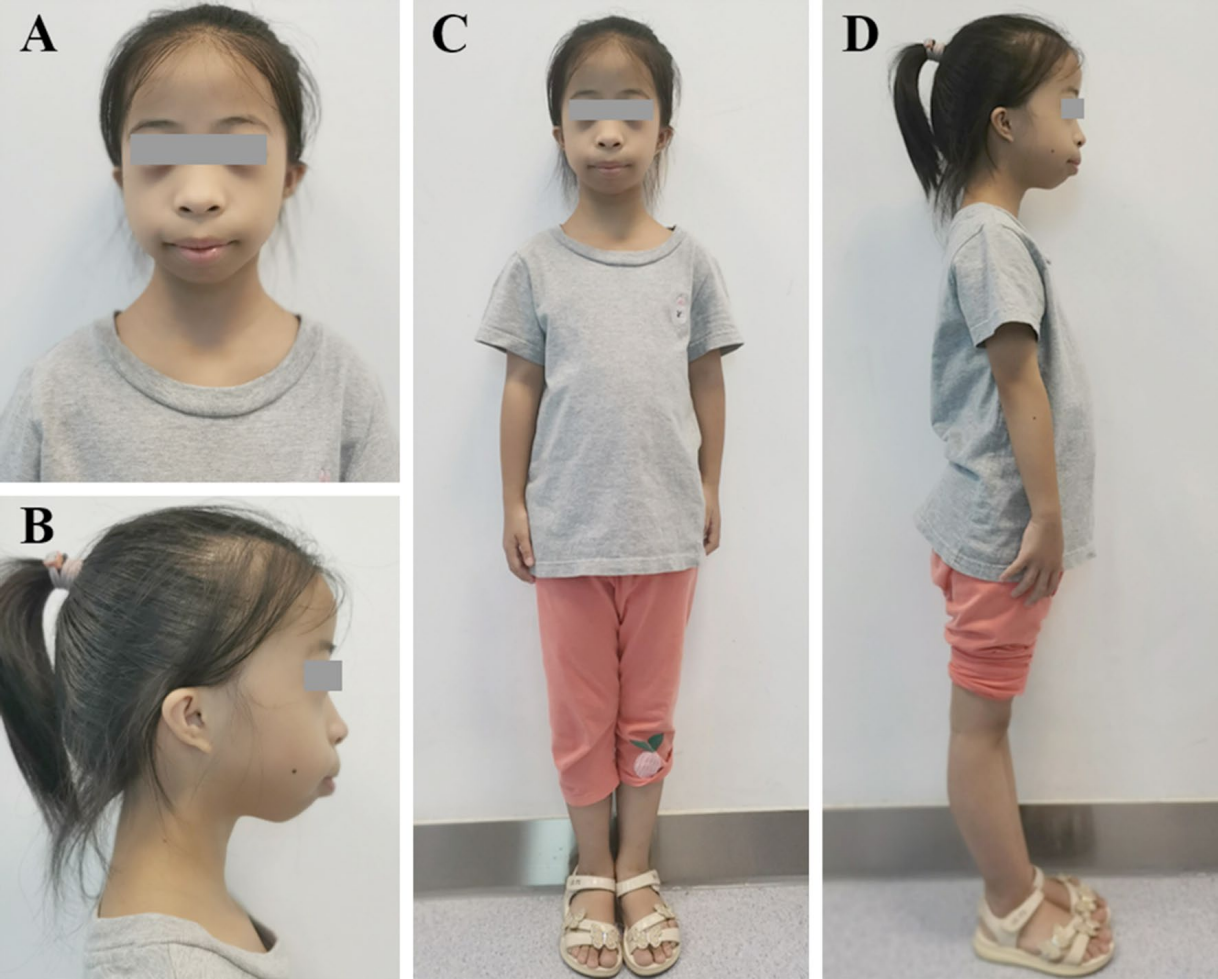
Clinical features of the patient. (A-D) The patient has typical Meier–Gorlin syndrome features, including a high forehead, microtia, low-set ears, a small mouth with full lips, retrognathia and micrognathia, knee hyperextension, and a proportionately short stature

A 4-year-old girl visited the paediatric endocrinology clinic of Tongji Hospital due to severe short stature, with a growth velocity of 4.0 cm/year. She was born by caesarean section at 38 weeks of gestation from nonconsanguineous parents. Foetal growth restriction was detected by routine ultrasound screening. The patient was born small for gestational age (SGA), with a birth weight of 1.98 kg (-3.1 SDS) and a birth length of 42 cm (− 4.5 SDS). She had feeding difficulties during the first year of life. At the first visit, her height was 86 cm (-4.4 SDS), her weight was 10 kg (-3.2 SDS), and her head circumference was 47.9 cm (-0.8 SDS). Dysmorphic features included a high forehead, small mouth with full lips, irregular tooth alignment, retrognathia and micrognathia, low-set ears and bilateral microtia, and hyperextended knees (Fig. 1). The fourth toe of the patient was shorter than the others, but her walking and motor abilities were normal. No abnormalities were found in the breast, external genitalia, or hearing. The MPH of the girl was calculated to be 156.5 cm (-0.8 SDS) based on the heights of her father (168 cm) and her mother (158 cm), respectively.

**Fig. 2:**
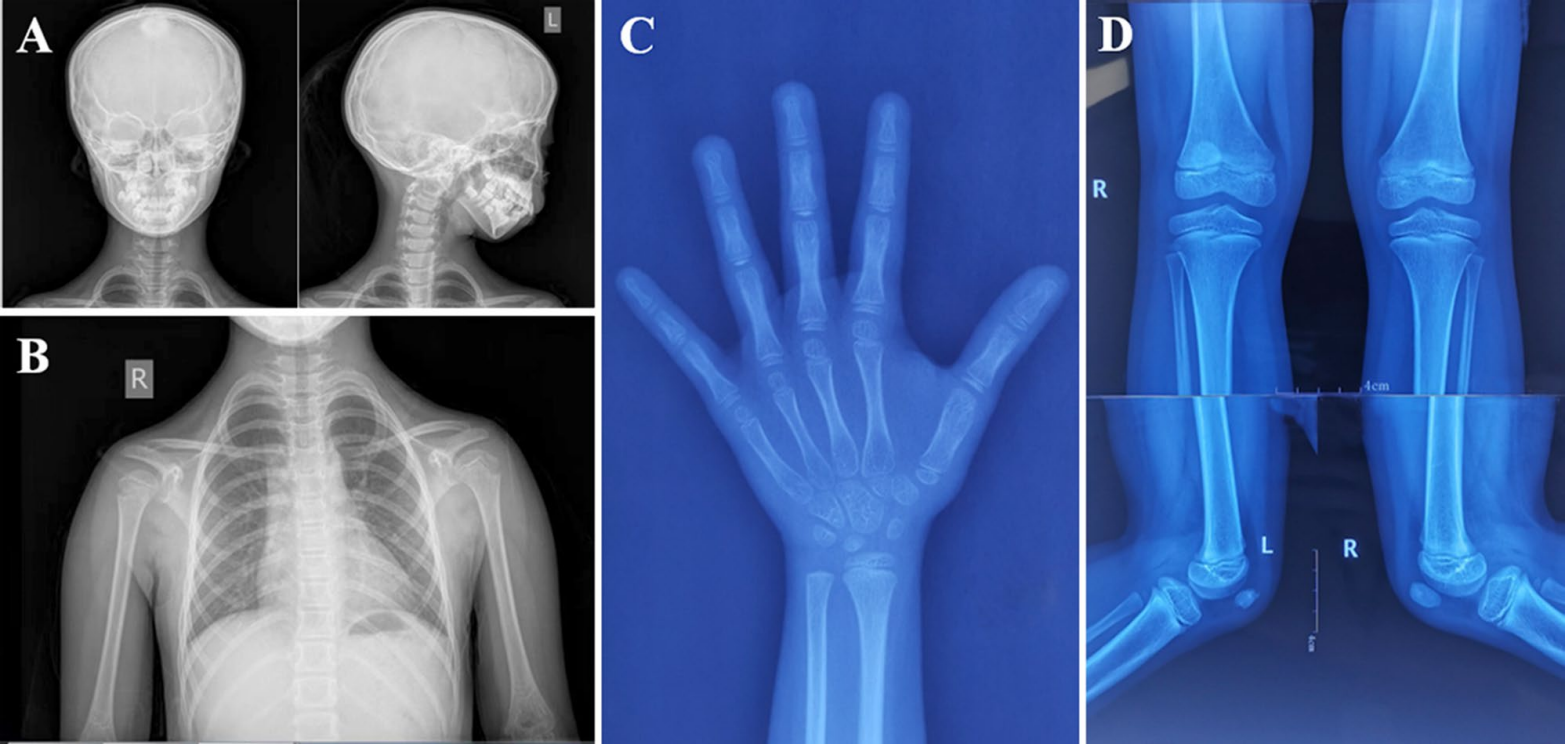
Imaging features of the patient. (**A**) Plain radiography of the skull showing micrognathia, retrognathia, and irregular tooth alignment. (**B**) Plain chest radiograph showing slender long bones and ribs. (**C**) Anteroposterior X-ray radiograph of the left hand at age 8. (**D**) Knee X-ray showing the left patella hypoplasia

X-ray examination revealed microretrognathia and irregular tooth alignment, slender humerus and ribs, and two-year-delayed bone age (the bone age was 6 years at the chronological age of 8 years) (Fig. 2**A**-**C**). A knee radiograph of the patient revealed left patellar hypoplasia at 8 years of age (Fig. 2**D**). GH stimulation tests revealed a peak GH concentration of 21.8 ng/mL. Endocrine examination revealed an IGF-1 level of 121 ng/mL and an IGFBP-3 level of 3970 ng/mL. Her adrenal function, thyroid function, and sex hormone levels were within the normal ranges, and no abnormalities were detected via cardiac ultrasound, abdominal ultrasound, or pituitary magnetic resonance imaging (MRI). MGORS was clinically suspected given her typical triad of microtia, hypoplastic patella, and intrauterine and postnatal growth retardation.

## Genetic and bioinformatic analysis

**Fig. 3:**
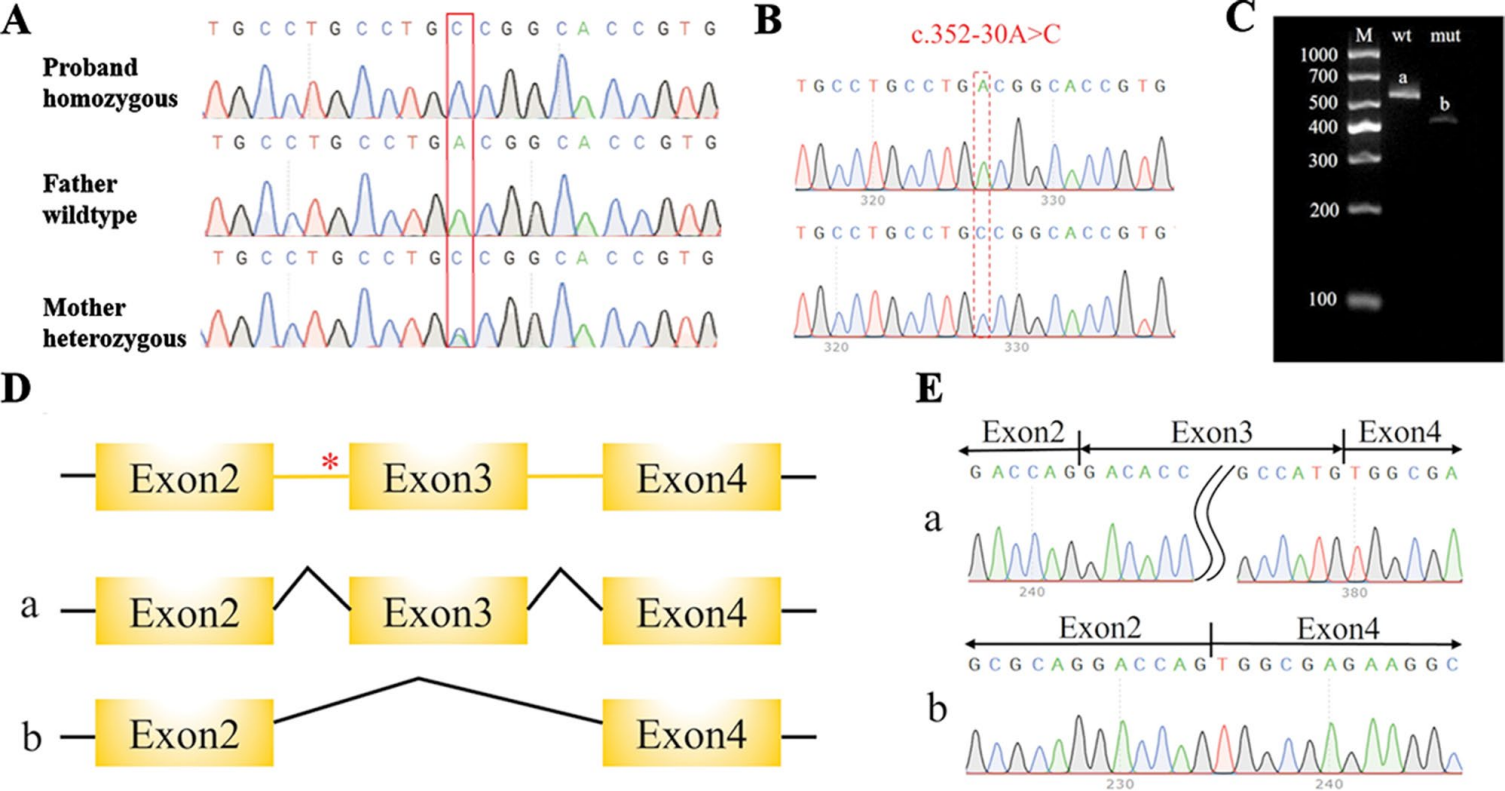
(**A**) Sanger sequencing of the CDT1 mutation and the sequencing chromatograms showing the homozygous A to C transition. (**B**) Sequencing map constructed by Minigene: the top is wt, and the bottom is mut. (**C**) Agarose gel of the RT–PCR products from HEK–293T cells transfected with plasmids containing either wt (band a) or mut (band b). (**D**) Schematic diagram of the minigene construction strategy and splicing, with the red * indicating the mutation position. (**E**) Corresponding sequencing result map of splicing bands

In conjunction with the clinical data from the patient, WES was performed on the patient and her parents to investigate the underlying causes of her severe short stature. The analysis identified a homozygous intronic variant, c.352–30 A > C, located in intron 2 of the *CDT1* gene, which was confirmed by Sanger sequencing. No variants in *CDT1* were detected in her father; however, her mother was identified as a heterozygous carrier of the c.352–30 A > C variation (Fig. 3A). We employed computational algorithms to analyse the distribution of single-nucleotide polymorphisms (SNPs) on the basis of WES data from the patient’s family to detect potential regions of homozygosity (ROHs) [27; 28]. Based on ROHs that possibly correspond to uniparental disomy (UPD), we inferred that the patient’s homozygous variants are likely derived from maternal isodisomy. The *CDT1* c.352–30 A > C variant appears to be novel, as it has not been previously reported in population databases. Further investigation revealed that this intronic variant was located at a noncanonical splice site, and was predicted to disrupt the wild-type BP and affect splicing according to HSF software. This variant decreased the BP score by 31.79%, from 93.2 to 63.57. As *CDT1* is one of the most common genes with pathogenic variants causing MGORS, this intronic variant was likely responsible for the clinical phenotype of the patient.

### Functional study

Since this variant is a novel *CDT1* mutation, a minigene splicing assay was carried out to validate whether the intronic variant interferes with *CDT1* splicing. The exon 2–intron 2–exon 3–intron 3–exon 4 fragments were inserted into the pECMV vector to obtain pECMV-CDT1-wt/mut, which was subsequently transfected into HEK-293T cells to observe the splicing pattern of exon 2–exon 3–exon 4. The extracted RNA was reverse-transcribed into cDNA and amplified with specific primers for Sanger sequencing analysis. Analysis of band a from pECMV-CDT1-wt revealed that exons 2–4 formed completely mature mRNA via the splicing of all introns. In contrast, a single, shorter band b was detected from the pECMV-CDT1-mut construct, and sequencing analysis revealed exon 3 skipping (Fig. 3 B-E).

### GH treatment and follow-up

**Fig. 4:**
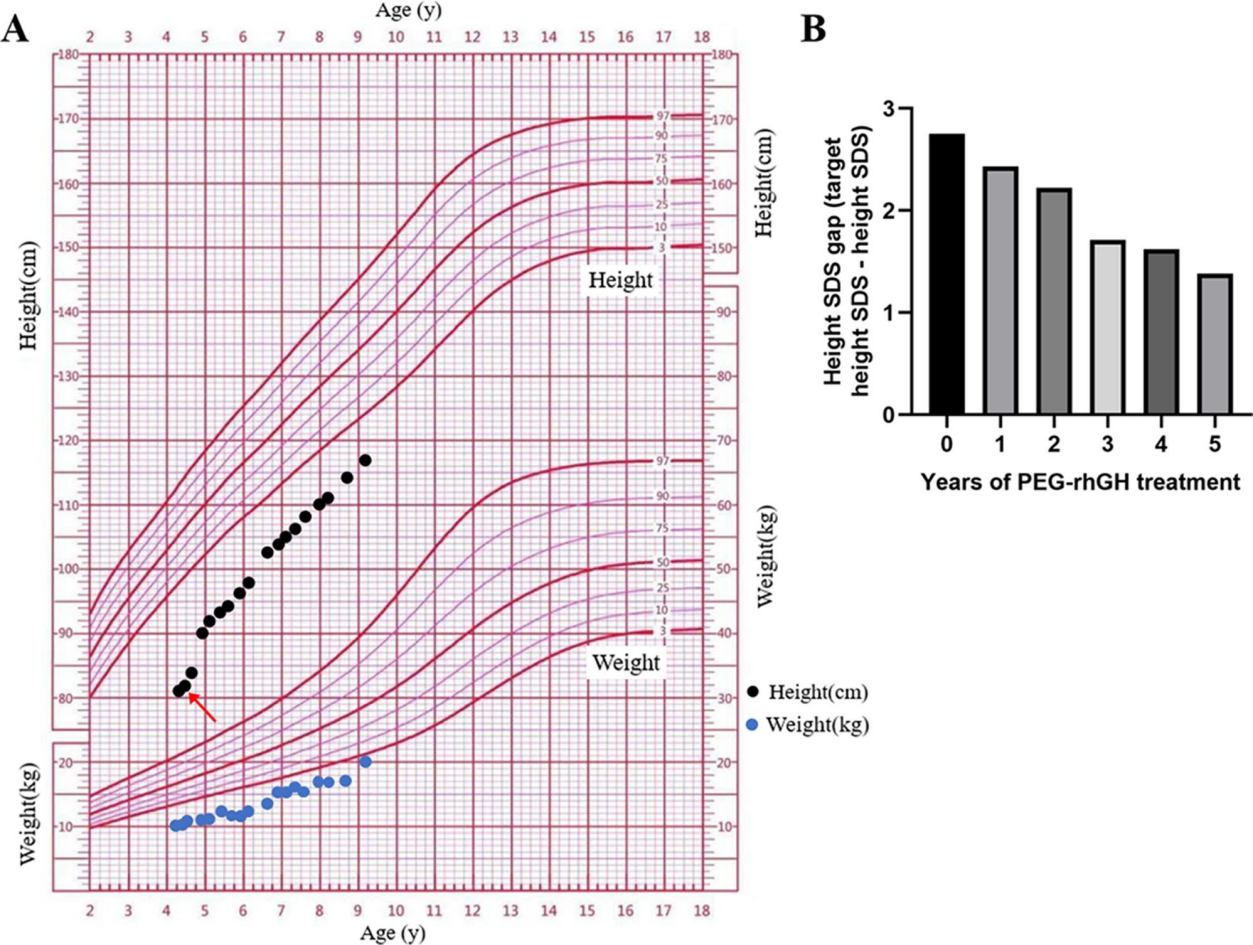
(**A**) The growth curve of the patient. The start of treatment with the long-acting PEGylated recombinant human growth hormone (PEG-rhGH) is shown by the red arrow. (**B**) The gap between the height SDS and the target height SDS of the patient during PEG-rhGH therapy

According to the clinical characteristics, the outcomes of exon sequencing and the minigene assay, the patient was clinically and molecularly diagnosed with MGORS. Given the severe prenatal and postnatal growth retardation, the girl was treated with once weekly PEG-rhGH for 5 years with the consensus of her parents. PEG-rhGH treatment was started when the patient was 4 years and 2 months old, with a body height of 86 cm (-4.4 SDS), a growth velocity of 4 cm/year, a bone age of 2 years, and an IGF-1 level of 121 ng/mL. The initial dosage of PEG-rhGH was 0.18 mg/kg/week. After the first year of PEG-rhGH treatment, her height increased to 93 cm (-4.0 SDS), and her growth velocity accelerated to 7.6 cm/year. The dosage of PEG-rhGH was adjusted to 0.2 mg/kg/week for the next 4 years according to her growth velocity. At the latest follow-up, the patient was 9 years and 2 months old with a bone age of nearing 8 years and an IGF-1 level of 373 ng/mL, and her body height reached 116.8 cm (-3.0 SDS) (Fig. 4A). Throughout the entire treatment period, her annual growth velocity accelerated from 4.0 cm/year to 6.2 cm/year, with a prominent increase of 1.4 in height SDS, and the gap between the height SDS and the target height SDS was shown in Fig. 4B. The thyroid function, fasting glucose, and fasting insulin were tested every 3–4 months during the treatment, and the results were all normal.

### Literature review of genetic variants

To gain further insight into the patterns of genetic variations and the associations between the mutated genes and height outcomes in patients with MGORS, we reviewed the reported literature on pathogenic variants of this rare disease. This analysis identified a total of 79 genetic variants, including the one we reported (Table 1). Among these variants, 12 were found in the PubMed database, 1 was found in the Chinese Journal of Medical Genetics, and the others were found in the HGMD.

**Table 1:** Overview of mutations identified in 13 genes related to MGORS

Gene	Nucleotide alterations	Amino-acid changes	Inheritance and zygosity	Variant effect	Variant site	Number of cases/ families	Variant reference ID
*ORC1*	c.266T > C	p.Phe89Ser	AR; Homozygous	Missense	Exon	1/1	CM112288
	[c.314G > A] + [c.1482–2 A > G]	p.Arg105Gln + intron 9 splice acceptor site	AR; Compound heterozygous	Missense + splice	Exon + intron	2/2	CM112289/CS112292
	c.1721 C > T	p.Thr57Met	Monoallelic	Missense	Exon	1/1	CM124031
	[c.314G > A] + [c.2159G > A]	p.Arg105Gln + p.Arg720Gln	AR; Compound heterozygous	Missense	Exon	1/1	CM112289/CM112290
	[c.314G > A] + [c.1999_2000delGTinsA]	p.Arg105Gln + p.Val667fsX24	AR; Compound heterozygous	Missense + frameshift	Exon	2/1	CM112289/CX112291
	c.380 A > G	p.Glu127Gly	AR; Homozygous	Missense	Exon	2/1	CM112287
	[c.314G > A] + [c.1996 C > T]	p.Arg105Gln + p.Arg666Trp	AR; Compound heterozygous	Missense	Exon	1/1	CM112289/CM112304
	[c.2483 C > T] + [c.2484delC]	p.Ser828Phe + p.Cys829ValfsTer30	AR; Compound heterozygous	Missense + frameshift	Exon	1/1	CM218431/CD218432
	[c1996C > T] + [c.692del]	p.Arg666Trp + p.Pro231Glnfs*12	AR; Compound heterozygous	Missense + frameshift	Exon	1/1	CM112304/CD210714
	c.721 + 1G > C	intron 5 splice donor site	Monoallelic	Splice	Intron	1/1	CS1515061
	c.2292 C > T	p.Ile764=	Monoallelic	Splice	Exon	1/1	35,282,325^1^
*ORC4*	[c.521 A > G] + [c.874_875insAACA]	p.Tyr174Cys + p.Ala292fsX19	AR; Compound heterozygous	Missense + frameshift	Exon	2/2	CM112293/CI112294
	[c.623 C > G] + [c.956 A > G]	p.Ser208X + p.His319Arg	AR; Compound heterozygous	Nonsense + missense	Exon	1/1	CM2112071/CM2112072
	c.521 A > G	p.Tyr174Cys	AR; Homozygous	Missense	Exon	4/3	CM112293
	[c.521 A > G] + CNV del	p.Tyr174Cys + del	AR; Compound heterozygous	Missense + deletion	Exon	1/1	CM112293/CG112306
*ORC6*	[c.2T > C] + [c.449 + 5G > A]	p.Met1? + p.?	AR; Compound heterozygous	Missense + splice	Exon + intron	4/3	CM124028/CS124033
	[c.257_258delTT] + [c.695 A > C]	p.Phe86X + p.Tyr232Ser	AR; Compound heterozygous	Nonsense + missense	Exon	3/1	CD112302/CM112301
	c.67 A > G	p.Lys23Glu	AR; Homozygous	Missense	Exon	1/1	CM174223
	c.71 C > T	p.Ala24Val	AR; Homozygous	Missense	Exon	1/1	CM2117278
	c.602_605delAGAA	p. Lys202Rfs*1	AR; Homozygous	Frameshift	Exon	1/1	CD151918
	c.712 A > T	p.Lys238X	AR; Homozygous	Nonsense	Exon	1/1	37,730,234^2^
*CDT1*	[c.196G > A] + [c.351G > C]	p.Ala66Thr + p.Gln117His	AR; Compound heterozygous	Missense + splice	Exon	1/1	CM112298/CM112303
	[c.1385G > A] + [c.1560 C > A]	p.Arg462Gln + p.Tyr520X	AR; Compound heterozygous	Missense + nonsense	Exon	4/2	CM112296/CM112297
	[c.351G > C] + [c.1385G > A]	p.Gln117His + p.Arg462Gln	AR; Compound heterozygous	Splice + missense	Exon	1/1	CM112303/CM112296
	[c.1081 C > T] + [c.1357 C > T]	p.Gln361X + p.Arg453Trp	AR; Compound heterozygous	Nonsense + missense	Exon	1/1	CM112299/CM112300
	[c.832G > T] + [c.1385G > A]	p.Glu278X + p.Arg462Gln	AR; Compound heterozygous	Nonsense + missense	Exon	2/1	CM124026/CM112296
	c.1402G > A	p.Glu468Lys	AR; Homozygous	Missense	Exon	1/1	CM112305
	c.1385G > A	p.Arg462Gln	Monoallelic	Missense	Exon	2/1	CM112296
	[c.1078_1080del] + [c.1276–24 A > G]	p.(Ala360del) + intron 8 branchpoint	AR; Compound heterozygous	Deletion + splice	Exon + intron	1/1	CD211848/CS211849
	[c.1144delT] + [c.1385G > A]	p.Leu382Trpfs*34 + Arg462Gln	AR; Compound heterozygous	Frameshift + missense	Exon	2/1	CD1717227/CM112296
	c.88,873,770 C > T	p.Arg453Trp	AR; Homozygous	Missense	Exon	1/1	32,843,414^3^
	c.352–30 A > C	intron 2 branchpoint	AR; Homozygous	Splice	Intron	1/1	-
*CDC6*	c.968 C > G	p.Thr323Arg	AR; Homozygous	Missense	Exon	1/1	CM112295
	[c.230 A > G] + [c.232 C > T]	p.(Lys77Arg) + p.(Gln78Ter)	AR; Compound heterozygous	Missense + nonsense	Exon	1/1	CM223644/CM223645
*CDC45*	[c.1 A > C] + [ c.1388 C > T]	p.Met1? + p.Pro463Leu	AR; Compound heterozygous	Missense	Exon	1/1	CM168040/27,374,770^4^
	[c.1270 C > T] + [c.1388 C > T]	p.Arg424X + p.Pro463Leu	AR; Compound heterozygous	Nonsense + missense	Exon	1/1	27,374,770^5^/27,374,770^4^
	c.791 C > A	p.Ser264Tyr	AR; Homozygous	Missense	Exon	1/1	CM168042
	c.1660 C > T	p.Arg554Trp	AR; Homozygous	Missense	Exon	3/2	CM168044
	[c.1388 C > T] + [c.1532delC]	p.Pro463Leu + p.Pro511Glnfs*36	AR; Compound heterozygous	Missense + frameshift	Exon	1/1	27,374,770^4^/27,374,770^6^
	[c.203 A > G] + [c.333 C > T]	p.Gln68Arg + p.Asn111	AR; Compound heterozygous	Splice	Exon	2/1	CS168047/CS168049
	c.[464 A > G; 961 C > A] + [c.1440 + 14 C > T]	p.[Glu155Gly; Pro321Thr] + splicing effect	AR; Compound heterozygous	Missense + splice	Exon + intron	1/1	27,374,770^7^/27374770^8/^CS168060
	[c.1541_1544del] + [c.630G > A]	p.(Lys514 Thrfs*10) + p.(Arg 210 = )	AR; Compound heterozygous	Frameshift + splice	Exon	2/1	CD216726/CS216727
	[c.326_329dup] + [c.1512 C > T]	p.Asn111Ilefs*11 + p.His504 =	AR; Compound heterozygous	Frameshift + splice	Exon	1/1	34,000,999^9^/CM218068
	[c.326_329dupTATA] + [c.1117 C > T]	p.V109fs + p.R373W	AR; Compound heterozygous	Missense	Exon	1/1	CI205249/ CM174984
	[c.686 C > A] + [c.1512 C > T]	p.Ser229X + p.His504 =	AR; Compound heterozygous	Missense + splice	Exon	1/1	CJMG^a^/CM218068
*GMNN*	c.16 A > T	p.Lys6X	AD; Heterozygous	Nonsense	Exon	1/1	CM1513647
	c.35_38delTCAA	p.Ile12Lysfs*4	AD; Heterozygous	Frameshift	Exon	1/1	CD1513648
	c.50 A > G	p.Lys17Arg	AD; Heterozygous	Missense	Exon	1/1	CM1513649
*MCM3*	c.2417 A > T	p.(Gln806Leu)	AR; Homozygous	Missense	Exon	1/1	33,654,309^10^
*MCM5*	[c.850_851delAG] + [c.1397 C > T]	p.(Arg284Glyfs*49) + p.Thr466Ile	AR; Compound heterozygous	Frameshift + missense	Exon	1/1	CD174507/CM174506
*MCM7*	[c.415 C > T] + [c.1616 A > G]	p.(Gln139*) + p.(Tyr539Cys)	AR; Compound heterozygous	Nonsense + missense	Exon	1/1	CM2121751/CM2121752
*DONSON*	c.631 C > T	p.Arg211Cys	AR; Homozygous	Missense	Exon	3/3	CM1916550
	[c.494T > C] + [c.607-36G > A]	p.Phe165Ser + intron 3 branchpoint	AR; Compound heterozygous	Missense + splice	Exon + intron	1/1	CM202233/CS202238
	[c.1634 C > T] + [c.809 A > G]	p.Pro545Leu + p.Tyr270Cys	AR; Compound heterozygous	Missense	Exon	1/1	CM202234/CM202241
	[c.670 C > T] + [c.809 A > G]	p.Pro224Ser + p.Tyr270Cys	AR; Compound heterozygous	Missense	Exon	1/1	CM202235/CM202241
	c.1297 C > T	p.Pro433Ser	AR; Homozygous	Missense	Exon	1/1	CM179298
*GINS2*	c.341G > T	p.(Arg114Leu)	AR; Homozygous	Missense	Exon	1/1	CM2212866
*GINS3*	[c.71 A > G] + [c.245G > A]	p.(Asp24Gly) + p.(Arg82Gln)	AR; Compound heterozygous	Missense	Exon	1/1	35,603,789^11^/35,603,789^12^
	c.70G > A	p.(Asp24Asn)	AR; Homozygous	Missense	Exon	3/1	CM2014861
	c.71 A > G	p.(Asp24Gly)	AR; Homozygous	Missense	Exon	3/3	35,603,789^11^

AR, autosomal recessive; AD, autosomal dominant

^1–12^Variants were found in the PubMed database and are shown with a PubMed ID. ^a^The variant was found in the Chinese Journal of Medical Genetics (CJMG). Others were found in the HGMD and are shown with an HGMD access ID

A total of 79 pathogenic variants were identified across the 13 genes described above, involving a total of 88 cases. Among these genes, only *GMNN* was associated with autosomal dominant inheritance, and all other variants were linked to autosomal recessive inheritance. As shown in Table 1, the highest number of mutations was observed in the *ORC1*, *CDT1*, and *CDC45* genes, which were also involved in the largest number of cases. Moreover, compound heterozygous mutations were the most common type, followed by homozygous mutations, and 4 monoallelic mutations were found in *ORC1* and *CDT1*. The vast majority of the variants were located in exons, with only 7 variants found in introns. The primary effects of these variants on proteins were missense mutations, and other effects included splice site mutations, frameshift mutations, nonsense mutations, and deletions. Furthermore, we recorded the mutated genes and corresponding height characteristics of MGORS patients with known molecular defects (Table S2). Patients were found at ages ranging from 0 to 49 years, with heights between − 9.6 and − 0.2 SDS. Through a literature review, we found that body height was strongly affected by mutations in the *ORC1*, *CDC6*, and *GMNN* genes, with mean heights of -6.0 SDS, -5.6 SDS, and − 5.6 SDS, respectively. In contrast, mutations in *GINS2* (-0.6 SDS) and *MCM5* (-2.5 SDS) have a comparatively minor effect on height. Additionally, the mean height SDS for patients with *CDT1* mutations was − 3.6.

### Literature review of GH treatment

To explore whether GH therapy can increase height in children with MGORS, we reviewed the published literature and found that a total of 12 patients with MGORS were treated with GH [2–7], including the one we presented here (Table S3). At the initiation of GH treatment, the mean age of these patients was 3.7 ± 1.2 years (3 cases unknown), the mean height SDS was − 5.9 ± 1.2 (5 cases unknown), and the mean delayed bone age was 1.8 ± 0.7 years (6 cases unknown). Only 2 patients had low IGF-1 levels, but their stimulated GH levels were not low, at 11.4 and 26 mIU/L, respectively [3]. The mean duration of GH therapy was 4.8 ± 2.9 years. These data suggested that these children initiated GH therapy at a young age, presenting with significantly short stature, and the majority exhibited normal IGF-1 and stimulated GH levels. After treatment, a total of seven patients responded positively to GH therapy. Five of these patients presented increased height SDS, with an average increase of 2.2 ± 0.9. Two of the patients demonstrated an acceleration in growth velocity, with one increasing from 3 to 4 cm/year to 6–7 cm/year, and the other increasing from an unknown growth velocity to 8–10 cm/year. Based on the available data, 58% (7/12) of the MGORS patients showed a positive response to GH therapy, and no adverse reactions were reported among all the MGORS patients who received GH treatment.

Furthermore, we investigated whether the genotype of the MGORS patients was associated with the efficacy of GH treatment. Among the 12 patients, 11 had gene mutations: 3 in *ORC1*, 3 in *ORC4*, 2 in *CDT1*, 1 in *ORC6*, 1 in *CDC6*, and 1 in *GMNN*. Among the three patients with *ORC1* mutations, one exhibited a positive response to GH treatment, whereas the other two did not show a significant improvement in height. The responses to GH treatment in the three patients with *ORC4* mutations were comparable to those observed in the three patients with *ORC1* mutations. Among the two patients with *CDT1* variations, one responded positively to GH treatment, whereas the other did not demonstrate significant effects. Additionally, there was only one patient each with mutations in *ORC6*, *CDC6*, and *GMNN* who received GH treatment, and all three of these patients exhibited notable increases in height. These findings suggest that the response of MGORS patients to GH therapy may be partially related to specific molecular defects, but more cases are needed to further investigate the interactions among genotype, phenotype, and GH treatment outcomes.

## Discussion

In this study, we presented the case of a Chinese girl exhibiting the classic triad of MGORS. WES revealed a novel homozygous variant, c.352–30 A > C, located in intron 2 of the *CDT1* gene. This noncanonical splice-site variant has not been reported in population databases but is predicted to disrupt the normal branch point. A minigene splicing assay confirmed that the intronic variant c.352–30 A > C adversely affects the normal mRNA splicing of *CDT1* by causing the skipping of exon 3. Considering the clinical phenotype, genetic variation, and functional validation results, this variant can be classified as likely pathogenic according to the ACMG criteria (functional characterization: PS3, population data: PM2, and family phenotype highly specific for gene: PP4) [15]. With the consensus of her parents, the girl has been receiving treatment with PEG-rhGH for five years, which has demonstrated considerable effectiveness in alleviating her severe height deficiency.

MGORS is a rare genetic disorder with pathogenic variations in genes associated with DNA replication. The first step in the initiation of DNA replication starts from late mitosis (M) and early G1 phase, when the MCM2-7 helicase complex is loaded onto the replication initiation site with the facilitation of the licensing factors CDT1 and CDC6 [29]. The *CDT1* gene is located on chromosome 16q24.3 and contains 10 exons encoding a 546-amino-acid CDT1 protein. CDT1 is a fundamental protein ensuring that DNA replicates only once per cell cycle [30–32]. Pozo et al. [33] examined the *CDT1* mutations identified in MGORS patients and reported that these mutations led to cell proliferation defects, which resulted in growth deficiencies in these patients. According to our literature review, *CDT1* (with 13 variants) was one of the most mutated genes in patients with MGORS, and the others were *CDC45* (with 17 variants) and *ORC1* (with 13 variants). Additionally, patients with *CDT1* mutations accounted for the largest proportion of MGORS patients with specific molecular defects, approximately 19% (17/88). A study by Munnik et al. [2] revealed that among 35 MGORS patients with *ORC1*, *ORC4*, *ORC6*, *CDT1*, and *CDC6* mutations, 10 carried *CDT1* mutations, accounting for 29%. Notably, the lower percentage we acquired was most likely due to the expansion of pathogenic variants and the number of cases.

Furthermore, the pathogenic variants associated with MGORS are predominantly inherited in a compound heterozygous manner, followed by homozygous mutations. The majority of these variants are located in exons, with only a few exceptions reported in introns. To our knowledge, a total of seven cases of MGORS involving intronic variants have been documented; five of these were inherited in a compound heterozygous state, while one was monoallelic [2; 10; 34–37]. Only the variant we reported was a homozygous intronic mutation. Our novel genetic findings indicate that the homozygous intronic mutation of *CDT1* can also lead to MGORS, which extends the genetic spectrum of this rare disease.

Since 2009, WES has been commonly used as the primary technique for identifying the molecular basis of rare diseases because of its notably lower cost than that of whole-genome sequencing (WGS) [38]. However, WES analysis of non-exonic variants generally includes 2 base pairs of CSS variants, while variants in BPs and deep introns often remain overlooked [24; 39]. It has been reported that 80% of the BPs in annotated introns are located between 18 and 35 nucleotides upstream of the 3’ splice site [40]. The identified variant c.352–30 A > C is located at a BP. Seven of the 79 variants identified by our literature review were intronic variants, 4 of which were in CSSs, and 3 of which were in BPs. Our findings suggest that intronic variants are increasingly common in MGORS, especially at BPs. We propose that WES analysis should include more intronic regions upstream and downstream of exons, especially BPs and reported introns, to increase the molecular diagnostic rate of rare diseases.

To date, there is no standard or targeted treatment for individuals with MGORS, and the main treatment approaches are focused on ameliorating their symptoms. The growth retardation of MGORS patients beginning in utero and continuing through adolescence is well known. The mean birth length of these patients was − 3.9 SDS, and the mean birth weight was − 3.4 SDS [41]. For adult MGORS patients, the mean height SDS was − 4.5 SDS, with an average height of 137.7 cm for females and 147.0 cm for males [3]. In other words, infants with MGORS were born SGA, and they had no catch-up growth. Their height remained below the 3rd percentile, so it was important to improve their height outcomes. In this study, the patient was extremely short in height (-4.4 SDS), so PEG-rhGH was used to increase her height. The patient received 0.18–0.2 mg/kg/week long-acting rhGH treatment for 5 years. Her growth velocity accelerated from 4.0 cm/year to 6.2 cm/year, with a 1.4 SDS increase in height after PEG-rhGH therapy. This finding suggested a possible effect of PEG-rhGH treatment on the height outcomes of children with MGORS.

Munnik et al. [3] described 9 MGORS patients treated with GH and reported that GH therapy was effective in only four patients. Two of these patients had low IGF-1, so the researchers suggested that GH therapy should be considered for MGORS patients with low IGF-1. However, the two recently reported patients [4; 7] and the patient in our study had normal IGF-1 levels, and all showed an increase in height with GH treatment. Overall, the efficacy of GH therapy was 58% (7/12) in all MGORS patients receiving GH treatment, 100% (2/2) in those with low levels of IGF-1, and 50% (5/10) in those with normal levels of IGF-1. Our results are inconsistent with those of previous studies suggesting that children with MGORS with normal IGF-1 levels are unlikely to benefit from GH therapy [1; 3]. This discrepancy may be due to differences in the GH form, dosage, individual, and reactions to specific molecular defects. The limitations of the study include the limited number of patients with this rare disease, with even fewer patients treated with GH and incomplete clinical data. Therefore, more patients need to be diagnosed and started on GH therapy as early as possible to determine its efficacy and molecular mechanism in children with MGORS with normal IGF-1 levels.

## Conclusions

In conclusion, this study identified a novel homozygous intronic variant, c.352–30 A > C, in the BP site of *CDT1*. The mutation was confirmed to be pathogenic for MGORS, extending the genetic spectrum of this rare disease. Through a literature review, we investigated the variant spectrum of MGORS and explored the potential efficacy of GH treatment for increasing height in these patients. This research highlights the emerging role of homozygous intronic mutations in MGORS and provides more information for GH treatment of children with this disease.

## Electronic supplementary material

Below is the link to the electronic supplementary material.

Supplementary Material 1

## Data Availability

All supporting data are included in the article, further inquiries can be directed to the corresponding author.

## Abbreviations

MGORS: Meier-Gorlin syndrome
GH: Growth hormone
WES: Whole-exome sequencing
IGF-1: Insulin-like growth factor 1
CSS: lysine methyltransferases
SDS: Standard deviation score
PEG-rhGH: long-acting PEGylated recombinant human growth hormone
ACMG: American College of Medical Genetics and Genomics
IGFBP-3: Insulin-like growth factor binding protein-3
HGMD: Human Gene Mutation Database

## References

[1] de Munnik SA, Hoefsloot EH, Roukema J, Schoots J, Knoers NV, Brunner HG, Jackson AP, Bongers EM. Meier-Gorlin syndrome. Orphanet J Rare Dis. 2015;10:114. 10.1186/s13023-015-0322-x. 26381604 10.1186/s13023-015-0322-x PMC4574002

[2] de Munnik SA, Bicknell LS, Aftimos S, Al-Aama JY, van Bever Y, Bober MB, Clayton-Smith J, Edrees AY, Feingold M, Fryer A, et al. Meier-Gorlin syndrome genotype-phenotype studies: 35 individuals with pre-replication complex gene mutations and 10 without molecular diagnosis. Eur J Hum Genet. 2012;20:598–606. 10.1038/ejhg.2011.269. 22333897 10.1038/ejhg.2011.269 PMC3355263

[3] de Munnik SA, Otten BJ, Schoots J, Bicknell LS, Aftimos S, Al-Aama JY, van Bever Y, Bober MB, Borm GF, Clayton-Smith J, et al. Meier-Gorlin syndrome: growth and secondary sexual development of a microcephalic primordial dwarfism disorder. Am J Med Genet A. 2012;158A:2733–42. 10.1002/ajmg.a.35681. 23023959 10.1002/ajmg.a.35681

[4] Vakili R, Mobini M, Hatami F, Vakili S, Valizadeh N. Meier-Gorlin syndrome with prenatal ultrasound findings and successful growth hormone therapy: six years follow-up of a rare case. Radiol Case Rep. 2022;17:1512–20. 10.1016/j.radcr.2022.02.028. 35282325 10.1016/j.radcr.2022.02.028 PMC8904407

[5] Bongers EM, Fryer JMOA, Sarda P, Hennekam RC, Hall BD, Superneau DW, Harbison M, Poss A, van Bokhoven H, Hamel BC, Knoers NV. Meier-Gorlin syndrome: report of eight additional cases and review. Am J Med Genet. 2001;102:115–24. 11477602 10.1002/ajmg.1452

[6] Burrage LC, Charng WL, Eldomery MK, Willer JR, Davis EE, Lugtenberg D, Zhu W, Leduc MS, Akdemir ZC, Azamian M, et al. De Novo GMNN mutations cause autosomal-Dominant Primordial Dwarfism Associated with Meier-Gorlin Syndrome. Am J Hum Genet. 2015;97:904–13. 10.1016/j.ajhg.2015.11.006. 26637980 10.1016/j.ajhg.2015.11.006 PMC4678788

[7] Li J, Ding Y, Chang G, Cheng Q, Li X, Wang J, Wang X, Shen Y. A boy with Meier-Gorlin syndrome carrying a novel ORC6 mutation and uniparental disomy of chromosome 16. Zhonghua Yi Xue Yi Chuan Xue Za Zhi. 2017;34:68–72. 10.3760/cma.j.issn.1003-9406.2017.01.016. 28186598 10.3760/cma.j.issn.1003-9406.2017.01.016

[8] Vetro A, Savasta S, Russo Raucci A, Cerqua C, Sartori G, Limongelli I, Forlino A, Maruelli S, Perucca P, Vergani D, et al. MCM5: a new actor in the link between DNA replication and Meier-Gorlin syndrome. Eur J Hum Genet. 2017;25:646–50. 10.1038/ejhg.2017.5. 28198391 10.1038/ejhg.2017.5 PMC5437912

[9] Nabais Sa MJ, Miller KA, McQuaid M, Koelling N, Wilkie AOM, Wurtele H, de Brouwer APM, Oliveira J. Biallelic GINS2 variant p.(Arg114Leu) causes Meier-Gorlin syndrome with craniosynostosis. J Med Genet. 2022;59:776–80. 10.1136/jmedgenet-2020-107572. 34353863 10.1136/jmedgenet-2020-107572 PMC9340002

[10] Bicknell LS, Bongers EM, Leitch A, Brown S, Schoots J, Harley ME, Aftimos S, Al-Aama JY, Bober M, Brown PA, et al. Mutations in the pre-replication complex cause Meier-Gorlin syndrome. Nat Genet. 2011;43:356–9. 10.1038/ng.775. 21358632 10.1038/ng.775 PMC3068194

[11] Nielsen-Dandoroff E, Ruegg MSG, Bicknell LS. The expanding genetic and clinical landscape associated with Meier-Gorlin syndrome. Eur J Hum Genet. 2023;31:859–68. 10.1038/s41431-023-01359-z. 37059840 10.1038/s41431-023-01359-z PMC10400559

[12] Zabnenkova V, Shchagina O, Makienko O, Matyushchenko G, Ryzhkova O. Novel compound heterozygous variants in the CDC6 gene in a Russian patient with Meier-Gorlin Syndrome. Appl Clin Genet. 2022;15:1–10. 10.2147/TACG.S342804. 35023948 10.2147/TACG.S342804 PMC8747802

[13] Blakes AJM, Wai HA, Davies I, Moledina HE, Ruiz A, Thomas T, Bunyan D, Thomas NS, Burren CP, Greenhalgh L, et al. A systematic analysis of splicing variants identifies new diagnoses in the 100,000 genomes Project. Genome Med. 2022;14:79. 10.1186/s13073-022-01087-x. 35883178 10.1186/s13073-022-01087-x PMC9327385

[14] Anna A, Monika G. Splicing mutations in human genetic disorders: examples, detection, and confirmation. J Appl Genet. 2018;59:253–68. 10.1007/s13353-018-0444-7. 29680930 10.1007/s13353-018-0444-7 PMC6060985

[15] Richards S, Aziz N, Bale S, Bick D, Das S, Gastier-Foster J, Grody WW, Hegde M, Lyon E, Spector E, et al. Standards and guidelines for the interpretation of sequence variants: a joint consensus recommendation of the American College of Medical Genetics and Genomics and the Association for Molecular Pathology. Genet Med. 2015;17:405–24. 10.1038/gim.2015.30. 25741868 10.1038/gim.2015.30 PMC4544753

[16] Rivas MA, Pirinen M, Conrad DF, Lek M, Tsang EK, Karczewski KJ, Maller JB, Kukurba KR, DeLuca DS, Fromer M, et al. Human genomics. Effect of predicted protein-truncating genetic variants on the human transcriptome. Science. 2015;348:666–9. 10.1126/science.1261877. 25954003 10.1126/science.1261877 PMC4537935

[17] Vaz-Drago R, Custodio N, Carmo-Fonseca M. Deep intronic mutations and human disease. Hum Genet. 2017;136:1093–111. 10.1007/s00439-017-1809-4. 28497172 10.1007/s00439-017-1809-4

[18] Lord J, Gallone G, Short PJ, McRae JF, Ironfield H, Wynn EH, Gerety SS, He L, Kerr B, Johnson DS, et al. Pathogenicity and selective constraint on variation near splice sites. Genome Res. 2019;29:159–70. 10.1101/gr.238444.118. 30587507 10.1101/gr.238444.118 PMC6360807

[19] Zhang P, Philippot Q, Ren W, Lei WT, Li J, Stenson PD, Palacin PS, Colobran R, Boisson B, Zhang SY, et al. Genome-wide detection of human variants that disrupt intronic branchpoints. Proc Natl Acad Sci U S A. 2022;119:e2211194119. 10.1073/pnas.2211194119. 36306325 10.1073/pnas.2211194119 PMC9636908

[20] Li H, Ji CY, Zong XN, Zhang YQ. [Body mass index growth curves for Chinese children and adolescents aged 0 to 18 years]. Zhonghua Er Ke Za Zhi. 2009;47:493–8. 19951508

[21] Tanner JM, Goldstein H, Whitehouse RH. Standards for children’s height at ages 2–9 years allowing for heights of parents. Arch Dis Child. 1970;45:755–62. 10.1136/adc.45.244.755. 5491878 10.1136/adc.45.244.755 PMC1647404

[22] [Chinese guidelines for the diagnosis and treatment of pediatric growth hormone deficiency]. Zhonghua Er Ke Za Zhi. 2024;62:5–11. 10.3760/cma.j.cn112140-20230914-00183. 38154971 10.3760/cma.j.cn112140-20230914-00183

[23] Guo S, Wu S, Li Z, Huang L, Zhan D, Zhang C, Luo X. Clinical and functional characterization of a Novel mutation in AVPR2 causing nephrogenic diabetes insipidus in a four-Generation Chinese Family. Front Pediatr. 2021;9:790194. 10.3389/fped.2021.790194. 34956990 10.3389/fped.2021.790194 PMC8696154

[24] Jaganathan K, Kyriazopoulou Panagiotopoulou S, McRae JF, Darbandi SF, Knowles D, Li YI, Kosmicki JA, Arbelaez J, Cui W, Schwartz GB, et al. Predicting Splicing from primary sequence with deep learning. Cell. 2019;176:535–e548524. 10.1016/j.cell.2018.12.015. 30661751 10.1016/j.cell.2018.12.015

[25] Jian X, Boerwinkle E, Liu X. In silico prediction of splice-altering single nucleotide variants in the human genome. Nucleic Acids Res. 2014;42:13534–44. 10.1093/nar/gku1206. 25416802 10.1093/nar/gku1206 PMC4267638

[26] Zhou Y, Pozo PN, Oh S, Stone HM, Cook JG. Distinct and sequential re-replication barriers ensure precise genome duplication. PLoS Genet. 2020;16:e1008988. 10.1371/journal.pgen.1008988. 32841231 10.1371/journal.pgen.1008988 PMC7473519

[27] Del Gaudio D, Shinawi M, Astbury C, Tayeh MK, Deak KL, Raca G. Diagnostic testing for uniparental disomy: a points to consider statement from the American College of Medical Genetics and Genomics (ACMG). Genet Med. 2020;22:1133–41. 10.1038/s41436-020-0782-9. 32296163 10.1038/s41436-020-0782-9

[28] Bis DM, Schüle R, Reichbauer J, Synofzik M, Rattay TW, Soehn A, de Jonghe P, Schöls L, Züchner S. Uniparental disomy determined by whole-exome sequencing in a spectrum of rare motoneuron diseases and ataxias. Mol Genet Genomic Med. 2017;5:280–6. 10.1002/mgg3.285. 28546998 10.1002/mgg3.285 PMC5441426

[29] Rona G, Pagano M. CDT1, a licensing factor that limits rereplication. Mol Cell. 2023;83:1–3. 10.1016/j.molcel.2022.11.019. 36608666 10.1016/j.molcel.2022.11.019 PMC9923941

[30] Ratnayeke N, Baris Y, Chung M, Yeeles JTP, Meyer T. CDT1 inhibits CMG helicase in early S phase to separate origin licensing from DNA synthesis. Mol Cell. 2023;83:26–e4213. 10.1016/j.molcel.2022.12.004. 36608667 10.1016/j.molcel.2022.12.004 PMC7614657

[31] Li C, Tan YP, Ma XS, Wang ZB, Meng TG, Sun QY. CDT1 is the major functional regulatory subunit of the pre-replication complex in zygotes. Cell Prolif. 2023;56:e13377. 10.1111/cpr.13377. 36479743 10.1111/cpr.13377 PMC9977660

[32] Fujita M. Cdt1 revisited: complex and tight regulation during the cell cycle and consequences of deregulation in mammalian cells. Cell Div. 2006;1:22. 10.1186/1747-1028-1-22. 17042960 10.1186/1747-1028-1-22 PMC1621056

[33] Pozo PN, Matson JP, Cole Y, Kedziora KM, Grant GD, Temple B, Cook JG. Cdt1 variants reveal unanticipated aspects of interactions with cyclin/CDK and MCM important for normal genome replication. Mol Biol Cell. 2018;29:2989–3002. 10.1091/mbc.E18-04-0242. 30281379 10.1091/mbc.E18-04-0242 PMC6333176

[34] Farwell KD, Shahmirzadi L, El-Khechen D, Powis Z, Chao EC, Tippin Davis B, Baxter RM, Zeng W, Mroske C, Parra MC, et al. Enhanced utility of family-centered diagnostic exome sequencing with inheritance model-based analysis: results from 500 unselected families with undiagnosed genetic conditions. Genet Med. 2015;17:578–86. 10.1038/gim.2014.154. 25356970 10.1038/gim.2014.154

[35] Knapp KM, Murray J, Temple IK, Bicknell LS. Successful pregnancies in an adult with Meier-Gorlin syndrome harboring biallelic CDT1 variants. Am J Med Genet A. 2021;185:871–6. 10.1002/ajmg.a.62016. 33338304 10.1002/ajmg.a.62016

[36] Fenwick AL, Kliszczak M, Cooper F, Murray J, Sanchez-Pulido L, Twigg SR, Goriely A, McGowan SJ, Miller KA, Taylor IB, et al. Mutations in CDC45, encoding an essential component of the pre-initiation complex, cause Meier-Gorlin Syndrome and Craniosynostosis. Am J Hum Genet. 2016;99:125–38. 10.1016/j.ajhg.2016.05.019. 27374770 10.1016/j.ajhg.2016.05.019 PMC5005452

[37] Knapp KM, Sullivan R, Murray J, Gimenez G, Arn P, D’Souza P, Gezdirici A, Wilson WG, Jackson AP, Ferreira C, et al. Linked-read genome sequencing identifies biallelic pathogenic variants in DONSON as a novel cause of Meier-Gorlin syndrome. J Med Genet. 2020;57:195–202. 10.1136/jmedgenet-2019-106396. 31784481 10.1136/jmedgenet-2019-106396 PMC7042968

[38] Boycott KM, Rath A, Chong JX, Hartley T, Alkuraya FS, Baynam G, Brookes AJ, Brudno M, Carracedo A, den Dunnen JT, et al. International Cooperation to enable the diagnosis of all Rare Genetic diseases. Am J Hum Genet. 2017;100:695–705. 10.1016/j.ajhg.2017.04.003. 28475856 10.1016/j.ajhg.2017.04.003 PMC5420351

[39] Wang ET, Sandberg R, Luo S, Khrebtukova I, Zhang L, Mayr C, Kingsmore SF, Schroth GP, Burge CB. Alternative isoform regulation in human tissue transcriptomes. Nature. 2008;456:470–6. 10.1038/nature07509. 18978772 10.1038/nature07509 PMC2593745

[40] Taggart AJ, DeSimone AM, Shih JS, Filloux ME, Fairbrother WG. Large-scale mapping of branchpoints in human pre-mRNA transcripts in vivo. Nat Struct Mol Biol. 2012;19:719–21. 10.1038/nsmb.2327. 22705790 10.1038/nsmb.2327 PMC3465671

[41] Niklasson A, Albertsson-Wikland K. Continuous growth reference from 24th week of gestation to 24 months by gender. BMC Pediatr. 2008;8:8. 10.1186/1471-2431-8-8. 18307822 10.1186/1471-2431-8-8 PMC2294116

