# Supplementary material for: A novel homozygous intronic variant in CDT1 that alters splicing causes Meier–Gorlin syndrome, and a review of published mutations and growth hormone treatments
Source: Orphanet J Rare Dis. 2024 Dec 18;19:465. doi: 10.1186/s13023-024-03430-4 (PMC11715027; doi:10.1186/s13023-024-03430-4)
Supplement: Supplementary file 1 — Supplementary Material 1 [file 13023_2024_3430_MOESM1_ESM.docx]

**A novel homozygous intronic variant in *CDT1*** **that alters splicing causes Meier–Gorlin syndrome, and a review of published mutations and growth hormone tr****eatments**

**Table S1** Primers used for the construction of the minigene plasmid, including minigene fragment fishing, mutation sites, and restriction enzyme cleavage sites

| **Primer name** | **Primer sequence (5′-3′)** |
| --- | --- |
| 623-CDT1-F | CGGAAGACGAGAGCAAGGCT |
| 2029-CDT1-R | GGTACACGGTTTTGATCTGGCC |
| 22-CDT1-F | AATTTGGCGGGAACCGCG |
| 1784-CDT1-R | GGTACTTGTAGGGCAGCACGAG |
| pECMV-CDT1-KpnI-F | TGACGATGACAAGCTTGGTACCGTTTCCAGCCCCAGTACC |
| CDT1-mut-F | CTGCCTGCCTGCCTGCCGGCACCGTGTCCCC |
| CDT1-mut-R | GGGGACACGGTGCCGGCAGGCAGGCAGGCAG |
| pECMV-CDT1-EcoRI-R | GTGCTGGATATCTGCAGAATTCCTACGCATCATGTCCTGGAC |
| pcMINI-CDT1-BamHI-F | TCATGGGTAGGTACCCGGATCCGTGAGGGGCGTGGGGAGACT |
| pcMINI-CDT1-EcoRI-R | GTGCTGGATATCTGCAGAATTCCTGTCAGGGAGGGAGGAGCCTCATG |
| pcMINI-F | ACCATGACGGTGATTATAAAG |
| pcMINI-R | TTTAAACGGGCCCTCTAGACTGGTCATTCCGGCTCCG |

**Table S****2** Mutated genes and short stature characteristics of MGORS patients

| **Gene** | **Sex ratio M/F** | **Age**  **at examination** | **Average**  **age at examination** | **Height for**  **age**  **(SDS range)** | **Average height**  **SDS** |
| --- | --- | --- | --- | --- | --- |
| *ORC1* (14 individuals) | 5 M/8 F (1 U) | 0-47 y | 11.5 y | -4.5 to -9.6 | -6.0 |
| *ORC4* (8 individuals) | 2 M/6 F | 2-23 y | 10 y | -1.8 to -6.4 | -4.5 |
| *ORC6* (11 individuals) | 6 M/1 F (4 U) | 4.5-15.4 y | 10 y | -2.4 to -3.3 | -2.8 |
| *CDT1* (17 individuals) | 3 M/12 F (2 U) | 0.5-49 y | 10.5 y | -0.2 to -5.1 | -3.6 |
| *CDC6* (2 individuals) | 1 M/1 F | 1.9-7 y | 4.5 y | -4.1 to -7 | -5.6 |
| *CDC45* (15 individuals) | 6 M/8 F (1 U) | 0.4-25 y | 7.7y | -1.3 to -7.7 | -3.8 |
| *GMNN* (3 individuals) | 1 M/2 F | 2.8-17 y | 7.7 y | -3.9 to -6.8 | -5.6 |
| *MCM3* (1 individual) | 0 M/1 F | 5.7 y | 5.7 y | -2.9 | -2.9 |
| *MCM5* (1 individual) | 1 M/0 F | 4.7 y | 4.7 y | -2.5 | -2.5 |
| *MCM7* (1 individual) | 0 M/1 F | 18 y | 18 y | -5 | -5 |
| *GINS2* (1 individual) | 0 M/1 F | 7 y | 7 y | -0.6 | -0.6 |
| *GINS3* (7 individuals) | 3 M/4 F | 4-24 y | 12.1 y | -1.1 to -5.2 | -2.8 |
| *DONSON* (7 individuals) | 4 M/3 F | 1.6-29.8 y | 10.9 y | -3.5 to -5.3 | -4.4 |
| Total (88 individuals) | 32 M/48 F (8 U) | 0-49 y | 9.9 y | -0.2 to -9.6 | -4.1 |

M, male; F, female; U, unknown; SDS, standard deviation score.

**Table S3** Summary of growth hormone treatment in 12 patients with MGORS

| **Patient** | **Sex** | **Gene** | **Mutations** | **GH treatment start** | | | | | |  | | **End of GH therapy follow up** | | |
| --- | --- | --- | --- | --- | --- | --- | --- | --- | --- | --- | --- | --- | --- | --- |
|  |  |  |  | **Age**  **(years)** | **Height**  **(SDS)** | **Growth velocity** | **CA-BA** | **GH**  **level** | **IGF-1**  **SDS** | | **Age**  **(years)** | | **Height**  **(SDS)** | **Growth velocity**  **or SDS gain** |
| 1^a^ | M | *ORC1* | c.2292C>T | 2.1 | -4.9 | U | 2-1.5 | U | N | | 5 | | U | 8-10 cm/year |
| 2^b^ | M | *ORC6* | c.67A>G | 5.5 | U | 3-4 cm/year | U | U | N | | 11 | | -2.8 | 6-7 cm/year |
| 3^c^ | M | *GMNN* | c.35_38delTCAA | 3 | -6.8 | U | 11.5-10 | L | -4.6 | | 14 | | -3 | 3.8 SDS gain |
| 4^c^ | F | *ORC1* | [c.314G>A] +  [c.1482-2A>G] | 4.5 | -7.3 | U | U | N | -1.07 | | 6.1 | | -7.3 | No improvement |
| 5^c^ | F | *ORC4* | [c.521A>G] + [c.874_875insAACA] | 3.1 | -7.1 | U | 3-1.2 | N | U | | 10 | | -5.3 | 1.8 SDS gain |
| 6^c^ | F | *ORC4* | c.521A>G | U | U | U | U | U | U | | 15 | | -5.5 | No improvement |
| 7^c^ | F | *ORC4* | c.521A>G | U | U | U | U | U | U | | 15 | | -5.8 | No improvement |
| 8^c^ | M | *ORC1* | c.380A>G | U | U | U | U | N | N | | 4.5 | | -5.2 | No improvement |
| 9^c^ | M | *CDT1* | [c.1385G>A] + [c1560C>A] | 3.5 | U | U | U | U | U | | 7.5 | | -4.7 | No improvement |
| 10^c^ | M | *CDC6* | c.968C>G | 2.5 | -5 | U | 15-12.5 | U | -0.8 | | 7 | | -3 | 2 SDS gain |
| 11^c^ | M | U | U | 5.4 | -5.7 | U | 5.3-3 | L | -3.3 | | 7.4 | | -3.7 | 2 SDS gain |
| 12 | F | *CDT1* | c.352-30A>C | 4 | -4.4 | 4 cm/year | 8-6 | N | N | | 9 | | -3.0 | 6.2 cm/year |

M, male; F, female; U, unknown; N, normal; L, low; SDS, standard deviation score; CA, chronological age; BA, bone age; GH, growth hormone; IGF-1, insulin-like growth factor 1

^a,b^ Individuals 1 and 2 from the articles of Vakili et al. and Li et al. [4; 7], respectively.

^c^Individuals 3-11 from the article of Munnik et al. [3].
